# Development and Validation of a Prediction Model for the Cure of Peritoneal Dialysis-Associated Peritonitis: A Multicenter Observational Study

**DOI:** 10.3389/fmed.2022.875154

**Published:** 2022-04-26

**Authors:** Lingfei Meng, Liming Yang, Xueyan Zhu, Xiaoxuan Zhang, Xinyang Li, Siyu Cheng, Shizheng Guo, Xiaohua Zhuang, Hongbin Zou, Wenpeng Cui

**Affiliations:** ^1^Department of Nephrology, The Second Hospital of Jilin University, Changchun, China; ^2^Department of Nephrology, The First Hospital of Jilin University-The Eastern Division, Changchun, China; ^3^Department of Nephrology, Jilin Central Hospital, Jilin, China; ^4^Department of Nephrology, Jilin FAW General Hospital, Changchun, China

**Keywords:** peritoneal dialysis, peritoneal dialysis-associated peritonitis, clinical decision rules, nomogram, ESRD – end stage renal disease

## Abstract

**Aim:**

Peritoneal dialysis (PD)-associated peritonitis (PDAP) is a severe complication of PD. It is an important issue about whether it can be cured. At present, there is no available prediction model for peritonitis cure. Therefore, this study aimed to develop and validate a prediction model for peritonitis cure in patients with PDAP.

**Methods:**

Patients with PD who developed PDAP from four dialysis centers in Northeast China were followed up. According to the region of PD, data were divided into training and validation datasets. Initially, a nomogram for peritonitis cure was established based on the training dataset. Later, the nomogram performance was assessed by discrimination (C-statistic), calibration, and decision curves.

**Results:**

Totally, 1,011 episodes of peritonitis were included in the final analysis containing 765 in the training dataset and 246 in the validation dataset. During the follow-up period, peritonitis cure was reported in 615 cases from the training dataset and 198 from the validation dataset. Predictors incorporated in the final nomogram included PD duration, serum albumin, antibiotics prior to admission, white cell count in peritoneal dialysate on day 5 (/μl) ≥ 100/μl, and type of causative organisms. The C-statistic values were 0.756 (95% CI: 0.713–0.799) in the training dataset and 0.756 (95% CI: 0.681–0.831) in the validation dataset. The nomogram exhibited favorable performance in terms of calibration in both the training and validation datasets.

**Conclusion:**

This study develops a practical and convenient nomogram for the prediction of peritonitis cure in patients with PDAP, which assists in clinical decision-making.

## Introduction

End-stage renal disease (ESRD) is a condition characterized by a high mortality rate and reduced quality of life, and patients receiving dialysis have experienced tremendous burdens (1). Peritoneal dialysis (PD) is a home-based and cost-effective dialysis modality for patients with ESRD, which can be customized depending on the value, expectation, and lifestyle of patients (2). PD has been increasingly utilized in many countries over the past decade (3), and China is becoming the country with the largest number of patients with PD globally (4). In light of the rapid spread of coronavirus disease 2019 (COVID-19) throughout the world, PD offers a great advantage over hemodialysis (HD) as an efficient renal replacement therapy with a low risk of coronavirus infection (5).

Peritoneal dialysis-associated peritonitis (PDAP) is a severe complication of PD. The incidence rate and outcome of peritonitis vary greatly across different countries (2). Despite the application of appropriate antibiotic therapy, PDAP still influences the mortality and technological survival rates of patients and contributes to the added hospitalization events and treatment costs (6, 7). Additionally, severe peritonitis episodes may progress to encapsulating peritoneal sclerosis, which precludes successful PD (8). It is a crucial clinical issue for clinicians to obtain a better outcome for this patient population. If individual peritonitis cure can be predicted based on the comprehensive assessment of the patient’s condition, a precise therapy and care strategy can be implemented to minimize patient suffering and save medical resources.

Several prognostic factors for PDAP cure have been reported in studies, including modifiable risk factors and non-modifiable risk factors (9, 10). However, to our knowledge, there are currently few available prediction models for the outcome of peritonitis. Therefore, this study aimed to establish and validate a prediction nomogram model in order to guide clinical decision-making and enhance the quality of PDAP treatment.

## Materials and Methods

### Study Participants

All patients with PD who developed PDAP during the study period from 01 January 2013 to 31 December 2019 were included and followed up in this study. Altogether 1,145 PDAP episodes were collected from four dialysis centers in Northeast China (Second Hospital of Jilin University, First Hospital of Jilin University, Jilin FAW General Hospital, and Jilin Central Hospital). The diagnosis of PDAP was made based on the presence of any two of the following features: (1) clinical features consistent with peritonitis, i.e., abdominal pain and/or cloudy dialysis effluent; (2) white cell count in dialysis effluent > 100/μl after a dwell time of at least 2 h, with > 50% polymorphonuclear leucocytes; and (3) positive culture of dialysis effluent (6). Meanwhile, the patient exclusion criteria were presented as follows: (1) those whose medical records were incomplete, (2) those younger than 18 years old, (3) those whose dialysis effluent was not cultured, (4) those who were treated with immunosuppressant medications, and (5) those with fungal or tuberculous peritonitis. According to the different regions of patients with PDAP, data were divided into training (First Hospital of Jilin University, Jilin Central Hospital) and validation (Jilin FAW General Hospital, Second Hospital of Jilin University) datasets. In accordance with the guidelines set forth by the International Society for Peritoneal Dialysis (ISPD), PDAP was treated with standard antibiotics covering both Gram-positive and Gram-negative pathogens. Once the culture results and sensitivities were known, antibiotic therapy was adjusted to narrow-spectrum agents. Treatment with effective antibiotics lasted for 2–3 weeks according to the type of organism. PD catheters were removed in accordance with the ISPD peritonitis recommendation. Informed consent was waived due to the retrospective and non-interventional design of this study by the Ethics Committee of Second Hospital of Jilin University (No. 2020026).

### Data Collection

Clinical information, including prognostic factors associated with peritonitis cure in PD, was retrieved from the medical records of patients (10). For each episode of peritonitis, the patient’s age at the time of PDAP, gender, number of PDAP episodes, PD duration, antibiotics used prior to admission, 24-h urine volume, presence of protopathy, and comorbidities (e.g., hypertension, diabetes mellitus, or cardiovascular disease) was recorded. In addition, results of various laboratory tests, including white blood cell count, serum albumin, hemoglobin, blood urea nitrogen, serum creatinine, peritoneal dialysate white cell count on admission, peritoneal dialysate white cell count on day 5 (/μl) ≥ 100/μl, presence of causative organisms, and exit-site infection, were also recorded. Furthermore, the causative organisms in effluent samples were subcategorized into culture-negative organisms, Gram-positive [*Staphylococcus aureus*, coagulase-negative *Staphylococcus* (CNS), *Corynebacterium*, Enterococcus, others], Gram-negative (*Pseudomonas aeruginosa*, *Escherichia coli*, *Klebsiella pneumoniae*, Enterobacter species, and others), and polymicrobial organisms. Polymicrobial peritonitis was defined as the presence of two or more cultured pathogens in the PD solution. All biochemical measurements were completed by adopting the standard laboratory techniques.

### Outcome

The primary outcome of interest was peritonitis cure, which was defined as the absence of a subsequent peritonitis event (relapse or recurrence), PD catheter removal, or transfers to HD (deemed permanent transfer to HD, or temporary transfer with failure to return to PD within the modality switch date 84 days), or death within 50 days after the onset of a peritonitis episode (10).

### Statistical Analysis

Logistic regression was employed to determine the potential outcome predictors in the training dataset. Those variables associated with peritonitis cure at *P* < 0.05 through univariate analysis were entered into a multivariable model. Backward stepwise regression was then performed, and a retention criterion of *P* < 0.1 was used to identify candidate variables for our prediction model. *P*-value < 0.05 was taken as a cut-off point for statistical significance. Thereafter, a nomogram was constructed based on the multivariate logistic regression model to calibrate the probability estimates of peritonitis cure individually. In the nomogram model, categorical covariates were considered dummy variables. All variables incorporated in logistic regression were predictors with < 10% of missing values. Continuous variables were interpolated by mean or median in accordance with the type of distribution, whereas categorical variables were interpolated by mode.

Internal validation was accomplished in the entire training dataset. Moreover, the C-statistic value was calculated to assess the model discrimination using Stata, and the calibration curve was subsequently plotted.

External validation was performed in the validation dataset based on the prediction model. The predicted value was obtained by employing the prediction function of Stata. Thereafter, the C-statistic value was calculated to assess the model discrimination, and the calibration curve was plotted. Moreover, decision curves were also plotted to evaluate the clinical usefulness of the nomogram.

Statistical analysis was carried out using SPSS (version 22.0, IBM, New York, NY, United States) and Stata (version 15.0; StataCorp LP) software. A *P*-value < 0.05 was considered statistically significant.

## Results

### Characteristics of Study Participants

**FIGURE 1:**
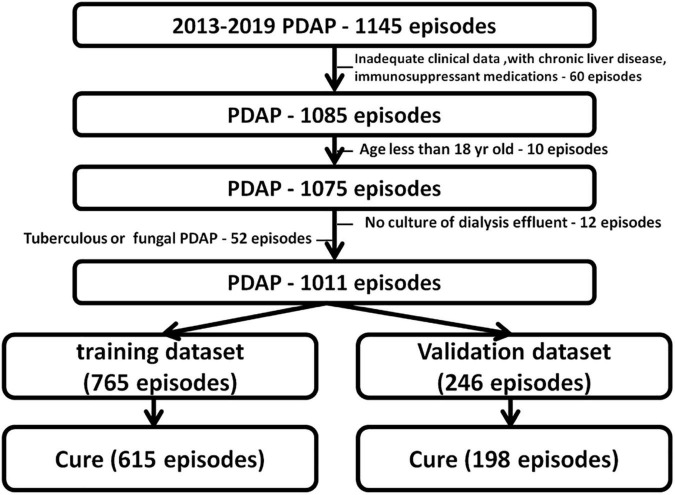
Flowchart of cohort establishment.

During the study period, 1,145 episodes of peritonitis from all four centers met the criteria for PDAP. Among these candidate participants, 60 were excluded due to missing data and immunosuppressant medications. Additionally, 10 were further eliminated for being younger than 18 years of age, 12 for no culture, and 52 for fungi or mycobacterium tuberculosis of dialysis effluent (Figure 1). Therefore, 1,011 episodes of PDAP were finally included for analysis. Among them, 813 episodes were cured, yielding an overall cure rate of 80.4%. There were 765 episodes in the training dataset and 246 in the validation dataset. During the follow-up period, 615 and 198 cases of peritonitis cure were observed in the training and validation datasets, respectively. Patient demographics, clinical manifestations, and laboratory parameters in the training dataset and validation dataset recorded at baseline are presented in Table 1. Patients in the training dataset were older, had a longer PD duration, and were inclined to not use antibiotics before admission, along with a less 24-h urine volume, a higher burden of cardiovascular disease and hypertension, higher blood urea nitrogen, peritoneal dialysate white cell count on admission, and peritoneal dialysate white cell count on day 5 (/μl), whereas a lower serum albumin level compared with the validation dataset (*P* < 0.05). There also existed significant differences in the pathogenic bacteria between the two datasets (*P* < 0.05).

**TABLE 1:** Baseline demographic and laboratory parameters of 1011 PDAP episodes in the training and validation dataset.

Index	Training dataset (*n* = 765)	Validation dataset (*n* = 246)	*P*
**Demographic characteristics**			
Age (year)	60(48, 69)	55 (42, 67)	0.000
Gender (male, *n*,%)	370 (48.4)	134 (54.5)	0.096
Number of PDAP episodes	1 (1, 2)	1 (1, 2)	0.203
PD duration (year)	1.34 (0.51, 2.62)	1.01 (0.36,2.25)	0.010
Antibiotics before admission (yes)	69 (9.0)	60 (24.4)	0.000
24-h urine volume ≥ 500 ml (yes)	468 (61.2)	172 (69.9)	0.013
**Protopathy**			0.177
Glomerulonephritis	300 (39.2)	106 (43.1)	
Interstitial nephritis	33 (4.3)	18 (7.3)	
Diabetic nephropathy	190 (24.8)	76 (30.9)	
Hypertensive renal impairment	129 (16.9)	16 (6.5)	
Other	113 (14.8)	30 (12.2)	
**Hypertension**	682 (89.2)	178 (72.4)	0.000
**Diabetes**	266 (34.8)	91 (37.0)	0.526
**Cardiovascular disease**	297 (38.8)	25 (10.2)	0.000
**Laboratory test**			
WBC (10^12^/L)	8.38 (6.15, 11.32)	8.10 (6.40, 11.10)	0.551
Hemoglobin (g/L)	99 (83, 112)	98 (85, 110)	0.682
Albumin (g/dL)	28.50 ± 6.25	30.81 ± 5.87	0.000
Blood urea nitrogen (mmol/L)	15.79 (12.10, 19.97)	14.54 (10.50, 20.14)	0.049
Serum creatinine (μmol/L)	714.73 (543.00, 904.00)	748.81 (533.00, 976.00)	0.085
Peritoneal dialysate cell count on admission (/μL)	2291 (800, 5760)	1075 (370, 2560)	0.000
Peritoneal dialysate white cell count on day 5(/μL) ≥ 100/μL	313 (40.9)	70 (28.5)	0.000
**Organisms (*n*, %)**			0.000
Culture-negative	132 (17.3)	118 (48.0)	
Gram-positive			
*Staphylococcus aureus*	34 (4.4)	14 (5.7)	
CNS	190 (24.8)	42 (17.1)	
Corynebacterium	10 (1.3)	0 (0.0)	
Enterococcus	15 (2.0)	4 (1,6)	
Others	109 (14.2)	16 (6.5)	
Gram-negative			
Pseudomonas aeruginosa	11 (1.4)	2 (0.8)	
Escherichia coli	80 (10.5)	19 (7.7)	
Klebsiella pneumoniae	21 (2.7)	0 (0)	
Enterobacter species	18 (2.4)	12 (4.9)	
Others	70 (9.2)	12 (4.9)	
Polymicrobial	75 (9.8)	7 (2.8)	
ESI/tunnel infection	3 (0.4)	2 (0.8)	0.600

*PD, peritoneal dialysis; WBC, white blood cell; PDAP, peritoneal dialysis-associated peritonitis; CNS, coagulase-negative Staphylococcus; ESI, exit-site infection.*

### Model Establishment

**FIGURE 2:**
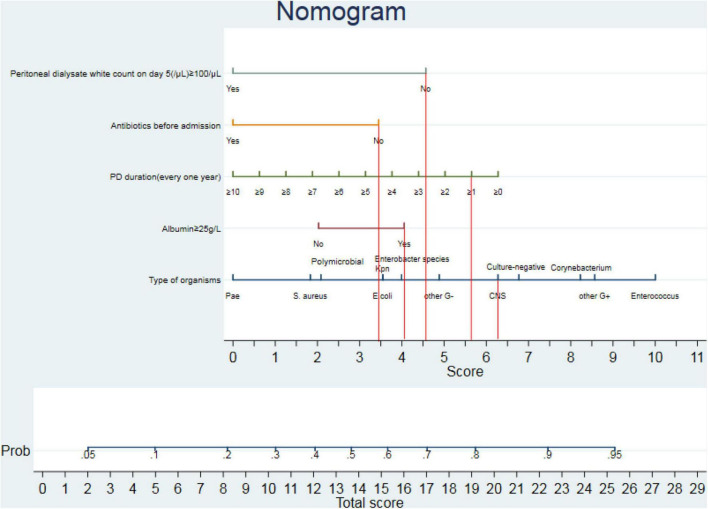
Nomogram for predicting the peritonitis-related catheter removal of peritoneal dialysis (PD)-associated peritonitis (PDAP). The nomogram provided a method to calculate the probability of peritonitis cure in patients with PDAP based on the combination of covariates in each patient. Its usage was illustrated with a 1-year history of PD, no antibiotics prior to admission, a serum albumin of 40 g/L, peritoneal dialysate white cell count on day 5 (/μl) < 100/μl, and CNS of causative organisms (vertical lines). The scores for PD duration, no antibiotics prior to admission, serum albumin, peritoneal dialysate white cell count on day 5 (/μl) < 100/μl, and bacterial infection for this patient were 5.7, 3.4, 4.1, 4.6, and 6.3 points, respectively, resulting in the total score of 24.1, which represented approximately 0.93 of cure probability. Pae, *Pseudomonas aeruginosa*; *S. aureus*, *Staphylococcus*; Kpn, *Klebsiella pneumoniae*; *E. coli*, *Escherichia coli*; CNS, coagulase-negative *Staphylococcus*.

In this study, the prediction model was established based on the training dataset. On univariate analysis, six variables [including PD duration, serum albumin, antibiotics prior to admission, peritoneal dialysate white cell count on day 5 (/μl) ≥ 100/μl, 24-h urine volume ≥ 500 ml, and type of causative organisms] were significantly associated with peritonitis cure (*P* < 0.05). Therefore, five predictors [including PD duration, peritoneal dialysate white cell count on day 5 (/μl) ≥ 100/μl, serum albumin, antibiotics prior to admission, and type of causative organisms] were incorporated into the final prediction model for multivariable analysis by backward selection (*P* < 0.05) (Table 2). A nomogram for the practical application of this model is shown in Figure 2. Its usage was illustrated with a hypothetical patient with a 1-year history of PD, no antibiotics prior to admission, serum albumin of 40 g/L, peritoneal dialysate white cell count on day 5 (/μl) < 100/μl, and CNS of causative organisms (Figure 2, vertical lines). The scores for PD duration, no antibiotics prior to admission, serum albumin, peritoneal dialysate white cell count on day 5 (/μl) < 100/μl, and bacterial infection for this patient were 5.7, 3.4, 4.1, 4.6, and 6.3 points, respectively, resulting in the total score of 24.1, which represented approximately 0.93 of cure probability. The nomogram assisted in the identification of patients with a high or low probability of cure. If it is intended to be cured, continuous maintenance of antibiotic therapy should be given. If it is intended not to be cured, early catheter removal and device insertion for temporary HD might be advised.

**TABLE 2:** Univariate and multivariable logistic regression of cure in the training dataset.

	Univariate			Multivariable		
Variable	B	OR (95% CI)	*P*	B	OR (95% CI)	*P*
PD duration (every 1 year)	–0.15	0.86 (0.79, 0.94)	0.001	–0.14	0.87 (0.79, 0.95)	0.003
Albumin ≥ 25 g/L	0.65	1.92 (1.32, 2.80)	0.001	0.51	1.67 (1.10, 2.54)	0.016
Antibiotics before admission	–0.73	0.48 (0.28, 0.83)	0.008	–0.87	0.42 (0.23, 0.77)	0.005
**Type of organisms**						
Culture-negative	Reference					
*Staphylococcus aureus*	–1.18	0.31 (0.13, 0.73)	0.007	–1.25	0.29 (0.12, 0.70)	0.006
CNS	–0.03	0.97 (0.52, 1.83)	0.932	–0.13	0.88 (0.46, 1.71)	0.711
Corynebacterium	0.41	1.51 (0.18, 12.64)	0.702	0.37	1.44 (0.17, 12.51)	0.739
Enterococcus	0.86	2.35 (0.29, 18.96)	0.421	0.82	2.26 (0.27, 18.87)	0.452
Other G +	0.51	1.67 (0.74, 3.75)	0.219	0.45	1.57 (0.67, 3.69)	0.296
Pseudomonas aeruginosa	–1.97	0.14 (0.04, 0.51)	0.003	–1.71	0.18 (0.05, 0.73)	0.016
Escherichia coli	–0.88	0.42 (0.21, 0.83)	0.012	–0.81	0.44 (0.21, 0.93)	0.031
Klebsiella pneumoniae	–1.09	0.34 (0.12, 0.94)	0.038	–0.81	0.44 (0.15, 1.32)	0.145
Enterobacter species	–0.83	0.44 (0.14, 1.37)	0.155	–0.70	0.50 (0.15, 1.66)	0.256
Other G-	–0.40	0.67 (0.31, 1.44)	0.307	–0.48	0.62 (0.28, 1.40)	0.250
Polymicrobial	–1.03	0.36 (0.18, 0.71)	0.003	–1.18	0.31 (0.15, 0.64)	0.002
24-h urine volume ≥ 500 ml	0.37	1,45 (1.01, 2.07)	0.045			
Peritoneal dialysate white cell count on day 5(/μL) ≥ 100/μL	–1.29	0.28 (0.19, 0.40)	0.000	–1.15	0.32 (0.21, 0.47)	0.000

*CNS: coagulase-negative Staphylococcus.*

### Model Validation

#### Internal Validation of the Prediction Model

**FIGURE 3:**
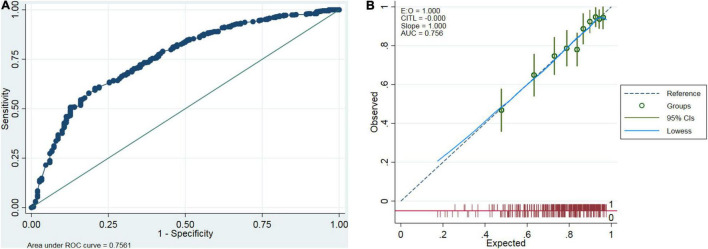
Internal validation of the prediction model. Discrimination was assessed by the receiver operating characteristic (ROC) curve **(A)**, and calibration was performed by the calibration curve **(B)** in the entire training dataset.

In the training dataset, the C-statistic value for the prediction of peritonitis cure by the constructed nomogram was 0.756 (95% CI: 0.713–0.799) (Figure 3A), indicating reasonable model discrimination. The calibration curve demonstrated that the probability of peritonitis cure predicted by the nomogram was relatively well matched with the actual measurement (Figure 3B).

#### External Validation of the Prediction Model

**FIGURE 4:**
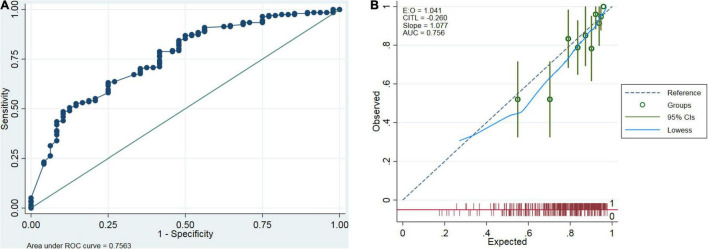
External validation of the prediction model. External validation was performed using the prediction model in the validation dataset. Discrimination was assessed by the ROC curve **(A)**, and calibration was completed by the calibration curve **(B)**.

**FIGURE 5:**
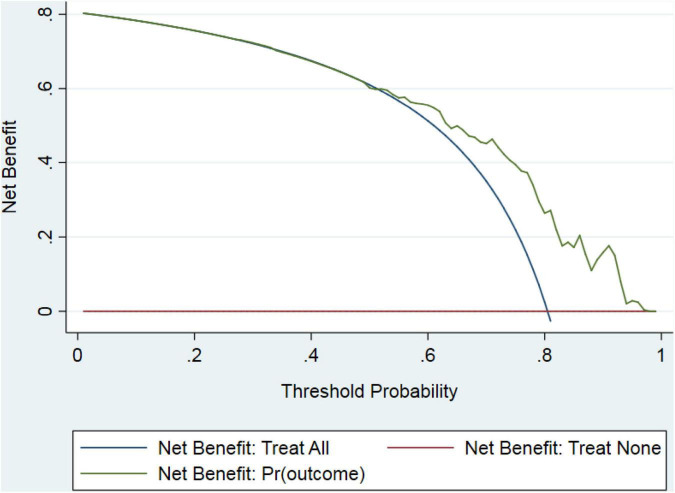
Decision curve for peritonitis-related catheter removal. Outcomes generated by the prediction model (green line) were distinct from those generated by “all” or “none” treatment strategies (blue or red lines), indicating that the use of the model might lead to improved clinical outcomes.

In the validation dataset, the C-statistic value for the prediction of peritonitis cure by the constructed nomogram was 0.756 (95% CI: 0.681–0.831) (the corresponding ROC curve is displayed in Figure 4A). In addition, the calibration curve exhibited good agreement between the nomogram-predicted value and the actual measurement (Figure 4B). A decision curve was also plotted to evaluate the clinical usefulness of the nomogram (Figure 5). Based on the above results, our nomogram accurately predicted patients with or without a cure.

## Discussion

A novel prediction model for peritonitis cure among patients with PDAP was established and validated in this multicenter study. PD duration, serum albumin, antibiotics prior to admission, peritoneal dialysate white cell count on day 5 (/μl) ≥ 100/μl, and type of causative organisms were included in the prediction model. According to our results, the as-constructed model showed good performance in calibration and discrimination, with a C-statistic value of over 0.75. Using the nomogram, it is possible to stratify individual peritonitis episodes and make reasonable treatment decisions. To the best of our knowledge, this is the first prediction nomogram proposed to predict PDAP cure in patients initializing PD in a multicenter study.

There are few reports concerning the prediction model of peritonitis cure in the PDAP population. Nochaiwong et al. developed a prediction score for the treatment failure among patients with PD, which incorporated DM, systolic blood pressure, dialysate white cell count on days 3–4, and dialysate white cell count on day 5 (11). Consistent with guidelines for the treatment of PDAP, we also included dialysate white cell count on day 5 in our prediction model (6). Nevertheless, their model did not incorporate pathogenic bacteria, and no external validation was conducted. Different from our study, their outcome was treatment failure containing catheter removal, transfer to HD, or peritonitis-associated mortality. In our study, the prediction nomogram model was more intuitive and applicable to clinical practice. The treatment decision should be made by taking into comprehensive consideration of a patient with PDAP.

According to our results, a shorter PD duration was related to the possibility of peritonitis cure, which was supported by several reports. For instance, a study found that a PD duration less than 2.4 years was associated with a higher resolution rate than that longer than 2.4 years (12). Another study indicated that a longer PD duration at the onset of peritonitis was associated with a longer duration from PD effluent abnormalities to treatment with appropriate antibiotics, which further led to adverse outcomes (13). A similar finding was also obtained from another study, which was that patients receiving long-term dialysis were prone to Gram-negative bacterial infection and had worse treatment outcomes compared with those undergoing short-term dialysis (14). There are inconsistent results regarding the impact of PD duration on the outcome of PDAP in the literature. For example, Yang et al. did not find any obvious relationship between PD duration and catheter loss (15). The differences may be ascribed to the different definitions of the study outcome. Our data confirmed that the increased PD duration reduced the probability of peritonitis cure in all the episodes of peritonitis. It can be inferred that continuous exposure to glucose and glucose degradation products may lead to tissue toxicity in the peritoneum, resulting in peritoneal dysfunction (16). This may further make it difficult to eliminate inflammation.

Another novel predictive factor for cure identified in this study was no antibiotics prior to admission. As far as we know, the relationship between antibiotics prior to admission and PDAP outcome remains unclear so far. The application of antibiotics at home may be related to serious patient conditions, which results in the low possibility of cure. Additionally, some patients applying antibiotics by themselves live far away from the PD center, and the remote distance from the hospital is also one of the risk factors for peritonitis and technique failure (17). Moreover, in our study, the application of antibiotics is not standardized at home by patients with PDAP, which delays the optimal timing of standard treatment (13) and adds to the difficulty in cure.

It was observed in this dataset that a higher level of serum albumin predicted a higher probability of peritonitis cure. In contrast to our study, one article considered that the serum albumin level did not influence the non-resolution of peritonitis (12). Another study reported that serum albumin was not the risk factor for the poor outcomes of patients with PDAP (18). Such differences may be attributed to their relatively small sample sizes that are insufficient to find the association between serum albumin and the outcome of PDAP. Hypoalbuminemia is identified as a risk factor for peritonitis in patients with PD (19–21). As reported in one study, hypoalbuminemia, a marker of malnutrition and inflammation, also predicted mortality in patients receiving PD (22). Moreover, a higher daily protein intake in patients with PD indicates a higher serum albumin level and good nutrition status, which prevents patient death or peritonitis (23). Theoretically, a higher serum albumin level has a good remedial effect when antibiotics are bound onto the serum albumin and the drug metabolism is reduced. We found that a high serum albumin level was good for the cure of PDAP, and it was assumed that treatment strategies to improve albumin levels should be advocated to improve the treatment outcome of peritonitis.

Noteworthily, the causative organism was included in the prediction model. Most studies classify pathogenic bacteria into several major categories. In the study conducted by Htay (24), the authors divided pathogenic bacteria into Gram-positive, Gram-negative, culture-negative, polymicrobial organisms, and others, finding that culture-negative bacteria had a higher cure rate than Gram-positive ones. Another study indicated that less virulent causative organisms (CNS, culture-negative, and Streptococci) were associated with a higher probability of cure (9). The prognosis of Gram-negative bacterial peritonitis was worse than that of Gram-positive bacterial peritonitis in Fung’s study (25). As a matter of fact, different bacteria in the same category may have different prognoses. Among Gram-positive bacterial infections, *S. aureus* peritonitis has a higher death rate than CNS peritonitis (26), which was recommended with a 3-week treatment in ISPD peritonitis recommendations. Among Gram-negative bacteria infections, *E. coli* peritonitis showed a poor prognosis of cure, transfer to hemodialysis, and death compared with non-*E. coli* Gram-negative peritonitis (25). We found that relative to culture-negative peritonitis, *S. aureus*, pseudomonas, *E. coli*, and polymicrobial peritonitis were associated with nominally lower odds of a cure. Although other bacteria did not reach statistical significance, we observed the trend from the nomogram. The different scores given by the bacterial types in the model fully explain the concrete effects of different bacteria on the cure. In this study, bacterial classification was more detailed, which overcame the problem of different prognoses of different Gram-positive or Gram-negative bacteria.

To the best of our knowledge, this is the first nomogram that provides clinicians with a predictable assessment tool for the cure of PDAP. Our research has a few strengths. First, our findings serve as a useful reference for the management of PDAP episodes by physicians, which relies on comprehensive assessment rather than a single factor. The external utility of the model is good and can be generalized. Timely ceasing PD can lower the length of hospital stay, medical costs, and occurrence of serious complications in patients with a low probability of PDAP cure. Furthermore, the nomogram is practical because all the variables included are easily and routinely collected clinical factors, offering an intuitive tool for individualized prediction using a small number of predictors.

Nonetheless, several limitations should also be pointed out in this study. First, given the retrospective nature of this study, there might be potential selection bias. Second, we did not consider new biomarkers such as IL-6, COX-2, RNase 3, and RNase 7 (27, 28). Future studies should develop or update the prediction model to include new biomarkers. In addition, larger population size and prospective investigations are also warranted.

## Conclusion

This study develops a practical and convenient nomogram with good accuracy in estimating the probability of peritonitis cure among patients with PDAP, which assists in clinical decision-making.

## Data Availability Statement

The raw data supporting the conclusions of this article will be made available by the authors, without undue reservation.

## Ethics Statement

The studies involving human participants were reviewed and approved by the Ethics Committee of Second Hospital of Jilin University (No. 2020026). The ethics committee waived the requirement of written informed consent for participation.

## Author Contributions

LFM explored the data and wrote this manuscript. XYL, SYC, SZG, and XHZ collected the data. LMY, XYZ, and XXZ provided the data. HBZ reviewed this manuscript. WPC designed this study and reviewed this manuscript. All authors contributed to the article and approved the submitted version.

## Conflict of Interest

The authors declare that the research was conducted in the absence of any commercial or financial relationships that could be construed as a potential conflict of interest.

## Publisher’s Note

All claims expressed in this article are solely those of the authors and do not necessarily represent those of their affiliated organizations, or those of the publisher, the editors and the reviewers. Any product that may be evaluated in this article, or claim that may be made by its manufacturer, is not guaranteed or endorsed by the publisher.
